# Animal use of fence crossings in southwestern rangelands

**DOI:** 10.1002/ece3.9376

**Published:** 2022-10-01

**Authors:** Lisa D. Zoromski, Randy W. DeYoung, John A. Goolsby, Aaron M. Foley, Jose A. Ortega‐Santos, David G. Hewitt, Tyler A. Campbell

**Affiliations:** ^1^ Caesar Kleberg Wildlife Research Institute Texas A&M University–Kingsville Kingsville Texas USA; ^2^ USDA Agricultural Research Service Cattle Fever Tick Research Laboratory Edinburg Texas USA; ^3^ East Foundation San Antonio Texas USA

**Keywords:** connectivity, crossings, fence, fence‐crossing, fencing, movement, rangelands

## Abstract

Net‐wire fencing built to confine livestock is common on rangelands in the Southwestern USA, yet the impacts of livestock fencing on wildlife are largely unknown. Many wildlife species cross beneath fences at defined crossing locations because they prefer to crawl underneath rather than jump over fences. Animals occasionally become entangled jumping or climbing over fences, leading to injury or death. More commonly, repeated crossings under net‐wire fencing by large animals lead to fence damage, though the damage is often tolerated by landowners until the openings affect the ability to enclose livestock. The usage, placement, characteristics, and passage rates of fence crossings beneath net‐wire fencing are poorly understood. We monitored 20 randomly selected fence crossings on net‐wire livestock fencing across two study sites on rangelands in South Texas, USA, from April 2018 to March 2019. We assessed the characteristics of fence‐crossing locations (openings beneath the fence created by animals to aid in crossing) and quantified crossing rates and the probability of crossing by all species of animals via trail cameras. We documented 10,889 attempted crossing events, with 58% (*n* = 6271) successful. Overall, 15 species of medium‐ and large‐size mammals and turkey (*Meleagris gallopavo*) contributed to crossing events. Crossing locations received 3–4 crossing attempts per day on average, but the number of attempts and probability of successful crossing varied by location and fence condition. The probability of crossing attempts was most consistently influenced by the opening size of the crossing and season; as crossing size (opening) increased, the probability of successful crossing significantly increased for all species. Peaks in crossing activity corresponded with species' daily and seasonal movements and activity. The density and size of fence‐crossing locations were dependent on fence maintenance and not associated with vegetation communities or habitat variables. However, crossing locations were often re‐established in the same locations after fence repairs. This is one of the few studies to monitor how all animal species present interacted with net‐wire livestock fencing in rangelands. Our results will help land managers understand the impact of net‐wire livestock fencing on animal movement.

## Graphical abstract

We assessed characteristics of fence‐crossing locations and quantified crossing rates and probability of crossing by all species of animals via trail cameras in South Texas. We documented 10,889 attempted crossing events. This is one of the few studies to monitor how all animal species present interacted with net‐wire livestock fencing in the southwestern rangelands.

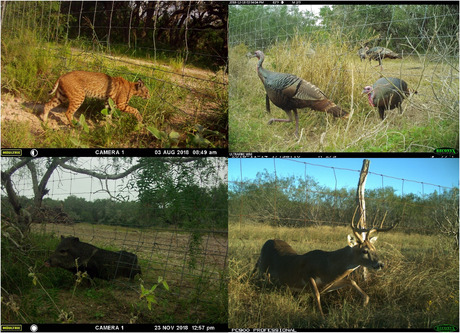

## 1 INTRODUCTION

Fencing is an anthropogenic feature that has been an integral tool to human society for millennia, and its use is common worldwide. Modern fences serve multiple purposes, such as marking property boundaries, confinement of livestock, and the reduction of trespassers (Hornbeck, 2010; Kotchemidova, 2008). Fence construction on private lands is often not regulated or even documented. As a result, the impact of fences on the landscape is often unknown and may vary temporally with the installation, removal, or repair of fencing (Jakes et al., 2018; McInturff et al., 2020). Although ecologists are beginning to appreciate the effects of anthropogenic linear features on wildlife, it is surprising that fences have received far less attention than roads or power lines (Jakes et al., 2018).

Fencing may directly impact wildlife if fences block access to water, shelter, or food (Harris et al., 2009; Williamson & Williamson, 1984). However, the indirect effects of fencing may have equal importance in the long‐term. For instance, fencing could intensify predation risk and impede animal escape from predation (Hölzenbein & Marchinton, 1992; ZhangQiang et al., 2013). Fence location can influence wildlife movement (Xu et al., 2021), and may funnel or entrap animals near interstate highways, increasing the risk of injury or mortality from vehicle collisions (Bellis & Graves, 1971; Harrington & Conover, 2006). In cases where wildlife exclusion is not an objective, it is important for animals to be able to safely cross barriers, such as livestock fencing, since animal populations benefit from connectivity (Crooks & Sanjayan, 2006).

Fence type, height, and size of openings under the fence influence the ease and method (jumping over or moving underneath) by which different species cross fences (Burkholder et al., 2018; Jones et al., 2018; Xu et al., 2021). Some individuals or species are reluctant or incapable of jumping over fences and prefer to cross underneath (Burkholder et al., 2018; Harrington & Conover, 2006). Fence height often determines an animal's willingness to jump (Burkholder et al., 2018; Jones et al., 2020; Thompson, 1978). Some species are more likely to cross under after modifications to existing fences, such as the addition of smooth (non‐barbed) bottom wire or clips to elevate the bottom wire (Burkholder et al., 2018; Jones et al., 2018).

Although the effects of fencing on wildlife are becoming more apparent, the literature on fence impacts on wildlife is biased in terms of the species impacted and types of fencing considered. For instance, McInturff et al. (2020) found that only 8% (37 of 446) of fence studies reviewed considered responses of multiple focal species. Most research on the unintentional effects of fences on wildlife focused on single species – typically large ungulates, long‐distance migrators, and animals that face mortality from fence entanglement (Harrington & Conover, 2006; Jakes et al., 2018; McInturff et al., 2020). The bias in taxa and fence type has resulted in a lack of information as to how non‐target species are impacted by fences and limits comparisons among studies (McInturff et al., 2020).

**FIGURE 1:**
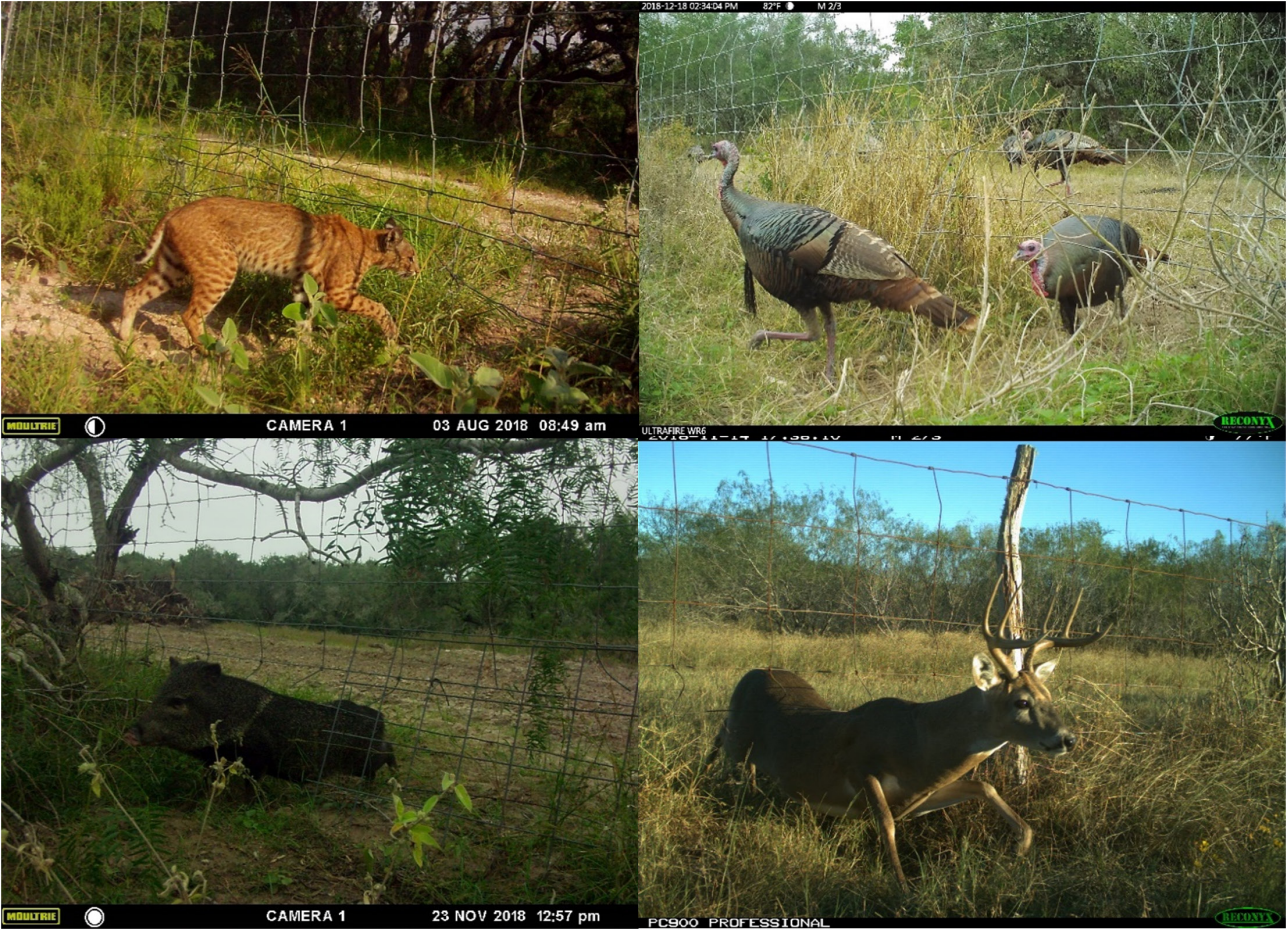
Bobcat, Turkey, collared peccary, and a white‐tailed deer passing beneath a net‐wire livestock fence at established crossing locations in South Texas, 2018. Repeated crossings by animals result in a recognizable opening and path under the fence, which can be enlarged if the back of the animal pushed up on the bottom wire of the crossing location.

Net‐wire livestock fencing is popular worldwide, but comparatively few studies have focused on the impacts of livestock fencing on wildlife (McInturff et al., 2020). Perceived as durable and easy to maintain (Isleib 1995), net‐wire fencing differs from other livestock fences, which tend to be constructed of barbed wire. Net‐wire fencing is an under‐appreciated source of mortality for many non‐target species, including upland birds (Baines & Summers, 1997; Bevanger & Brøseth, 2000; Catt et al., 1994; Robinson et al., 2016). Because many animals prefer to dig or push under rather than jump net‐wire fences, repeated crossing events result in the creation of crossing locations. The locations, termed “crossings,” or “crawl‐unders,” are recognized as locations where the bottom wire is pushed up or missing, often with a depression of bare soil acting as a path beneath (Figure 1). The term ‘fence crossing’ henceforth refers to passage underneath and not over the fence. Crossings are often created by animals that are strong enough to push up the bottom fence wire or to dig beneath. For instance, exotic species such as wild pigs (*Sus scrofa*) and nilgai antelope (*Boselaphus tragocamelus*) are known to create crossings in net‐wire fences by pushing up the bottom wire (Strickland et al., 2020; Zoromski, 2019). The openings are then used by other wildlife species (Weise et al., 2014).

In southwestern rangelands, it is common to have large areas managed for both livestock and native wildlife in grazing systems that use net‐wire livestock fences. Wildlife crossings under net‐wire fencing are common but lead to fence damage, which is often tolerated by landowners until the openings affect the ability to enclose livestock. However, the characteristics of wildlife fence crossings have not been quantified. There are no data on the density and opening size of crossings locations nor on the placement of fence‐crossing locations in relation to repaired locations and woody vegetation. Furthermore, information is lacking on the composition and frequency of animal species that use crossings, animal behavioral responses to crossings, and the timing of crossing events. The goal of our study was to gain information on how net‐wire livestock fencing impacts animal movements and behaviors in southwestern rangelands. Specific objectives were to (1) assess the density (crossing per linear m), the opening size of fence‐crossing locations, as well as their placement, and condition, and (2) quantify species‐specific probabilities of crossings relative to characteristics of fence crossing locations. We hypothesized a positive association between fence crossing size and body size of species, that the location of fence crossings may be associated with woody cover, and that crossing rates and probability of crossing correspond with species' seasonal activity patterns and relative abundance.

## 2 STUDY AREA

**FIGURE 2:**
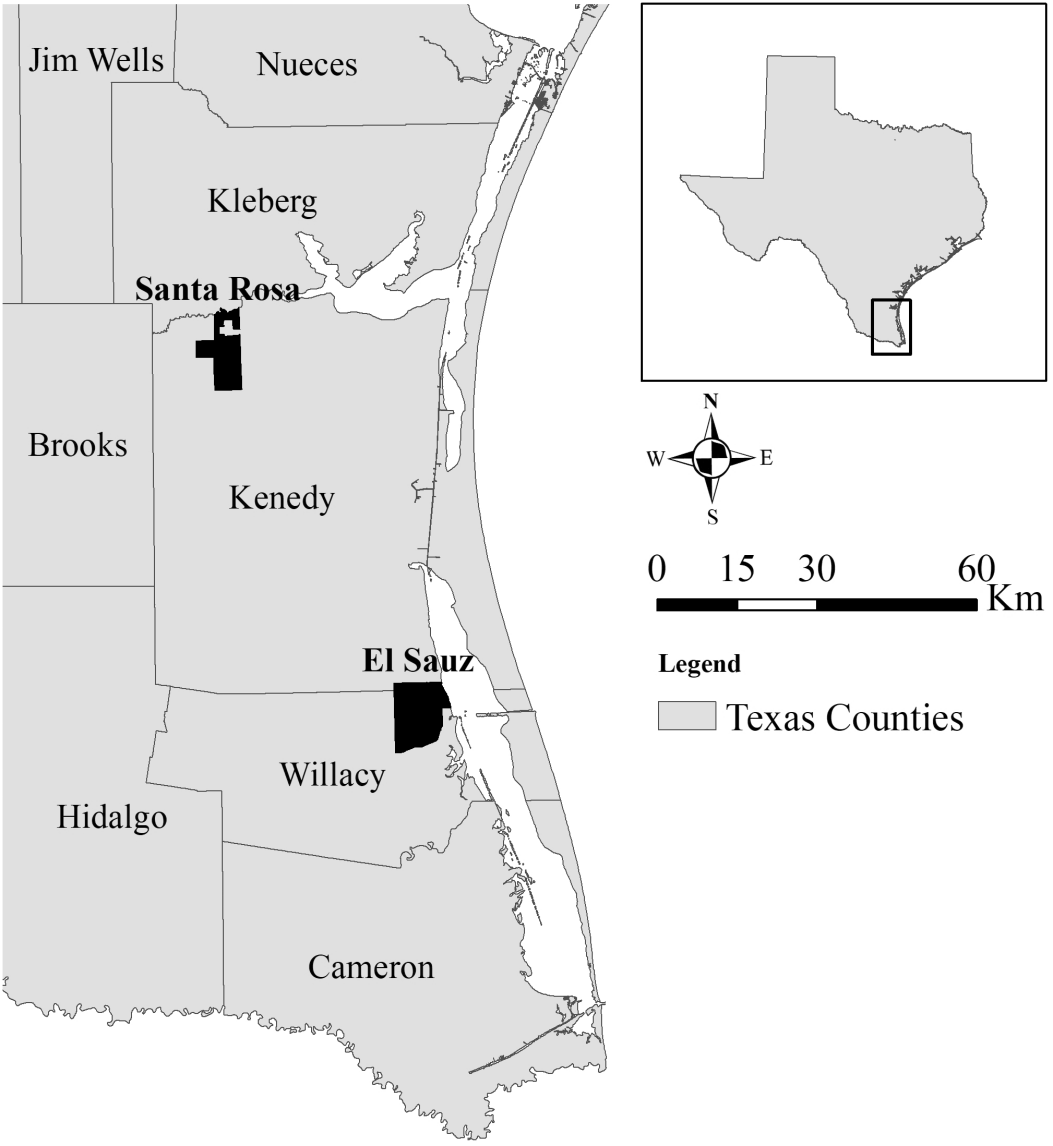
The Santa Rosa and El Sauz ranches in Kenedy and Willacy counties, respectively, in South Texas, USA. Camera traps were deployed on 10 randomly selected fence crossings at each site from April 2018–march 2019.

**FIGURE 3:**
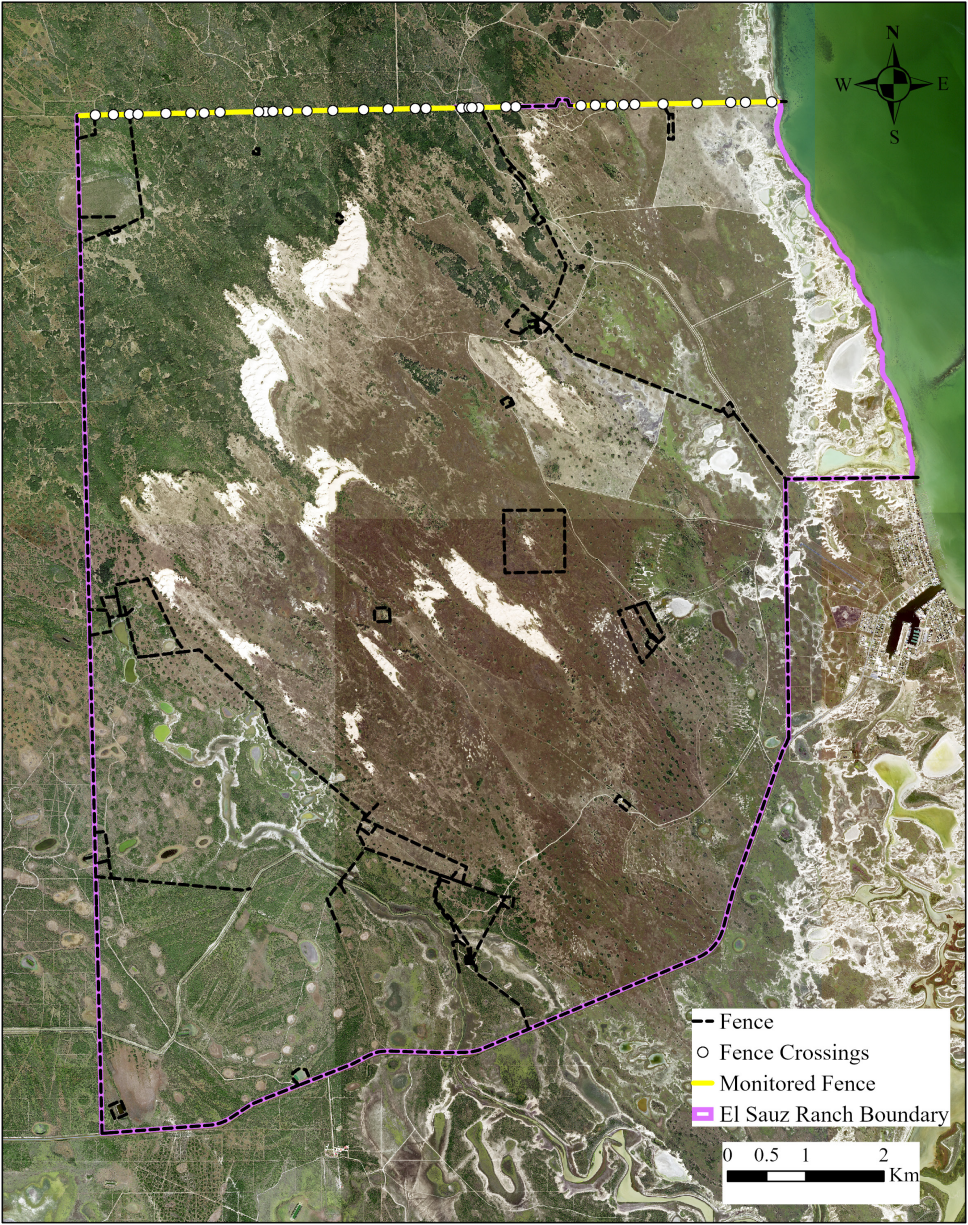
The El Sauz ranch in Willacy County, South Texas, USA. All fence crossings were recorded along the monitored fence, and camera traps were deployed over 10 randomly selected fence crossings from April 2018–march 2019.

**FIGURE 4:**
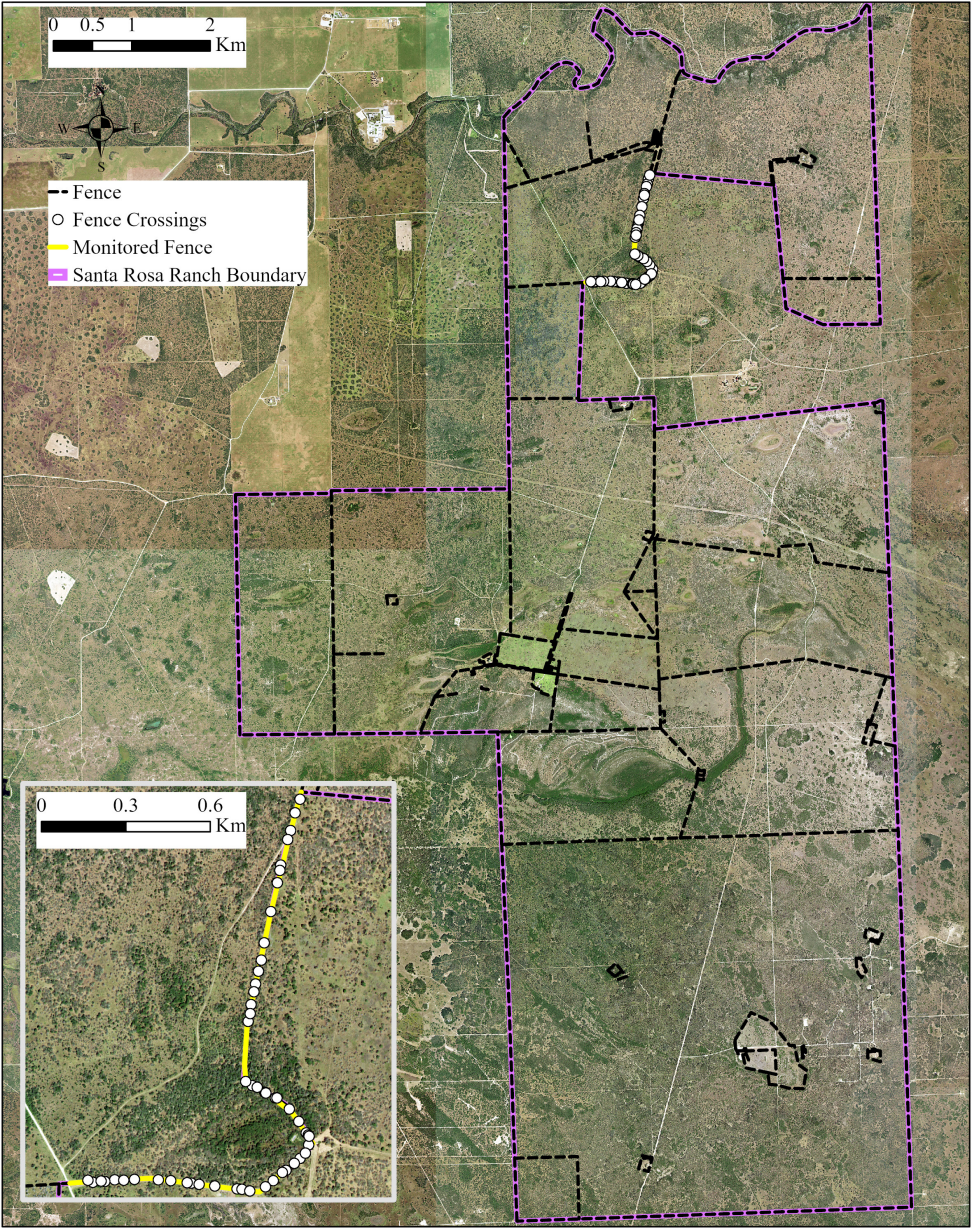
The Santa Rosa ranch in Kenedy County, South Texas, USA. All fence crossings were recorded along the monitored fence, and camera traps were deployed over 10 randomly selected fence crossings from April 2018–march 2019.

From April 2018 to March 2019, we studied fence line ecology at two sites in the South Texas region of the United States: the El Sauz and Santa Rosa Ranches (Figures 2, 3, 4). The sites are owned by the East Foundation, an Agricultural Research Organization that manages over 870 km^2^ of rangeland across South Texas, with the goal of promoting the advancement of land stewardship through ranching, science, and education (https://www.eastfoundation.net). The sites are maintained as native rangeland and working cattle ranches. Both sites have net‐wire livestock fencing for boundaries and dividing interior pastures with 0.31 × 0.20‐m mesh, ranging from 1.25 to 2 m in height.

The El Sauz Ranch is 10,984 ha and borders the community of Port Mansfield, Willacy County, Texas (26°40′N, 97°35′W). The area is located in the Coastal Sand Plain, Lower Rio Grande Valley, and Laguna Madre Coastal Marshes ecoregions (Bailey et al., 1994). The Coastal Sand Plain contains active sand dunes, closed‐depression ponds, and mid‐ to tall‐grass prairie. The Lower Rio Grande Valley ecoregion contains dense and diverse grassland, shrubland, and low woodland communities, with mostly Quaternary clay‐loams and sandy clay‐loam soils (WSS 2018). The Laguna Madre Coastal Marshes comprise a hypersaline lagoon system, interspersed with seagrass meadows and tidal mud flats (Bailey et al., 1994). Common vegetation communities include live oak (*Quercus virginiana*) woodlands, mesquite (*Prosopis glandulosa*) woodlands, gulf cordgrass (*Spartina spartinae*) grasslands, seacoast bluestem (*Schizachyrium scoparium* var. *littorale*) grassland, and marshhay cordgrass (*Spartina patens*) grassland. Port Mansfield had annual average precipitation of 64.3 cm and the average mean temperature of 23.2°C from 1998 to 2018 (Prism Climate Group [Prism] 2018).

The Santa Rosa Ranch is 7545 ha and is located near the community of Riviera, Kenedy County, Texas (27°13′N, 97°51′W). The area is located in the Coastal Sand Plain ecoregion (Bailey et al., 1994). Soils include Palobia loamy fine sand, Falfurrias‐Cayo complex, Sarita, Nueces, and Sauz fine sand, and Yturria fine sandy loam (WSS 2018). Dominant vegetation communities include mesquite woodlands, huisache (*Acacia farnesiana*) woodlands, live oak woodlands, and spiny aster (*Leucosyris spinosa*) wetlands. Riviera had annual average precipitation of 70.7 cm and an average mean temperature of 22.8°C from 1998–2018 (Prism Climate Group [Prism] 2018).

The East Foundation conducts annual aerial surveys for large mammals on each of its properties (Peterson et al., 2020). Surveys are completed by helicopter and population estimates are derived using distance sampling (Thomas et al., 2002). The Foundation monitors trends in population size for all large mammals detected, including exotic nilgai antelope and wild pigs, as well as native white‐tailed deer (*Odocoileus virginianus*), and collared peccary (*Pecari tajacu*). Nilgai, native to India and Pakistan (Dinerstein, 1980; Mirza & Khan, 1975), were introduced to the region during the 1930s and have expanded into a large, free‐ranging, population estimated at >30,000 individuals throughout the coastal South Texas region (Leslie, 2008; Traweek & Welch, 1992). Aerial surveys found deer and nilgai to be present on both the El Sauz and Santa Rosa sites (Annala, 2015; Peterson et al., 2020, East Foundation, unpublished data). In 2018 and 2019, respectively, survey estimates were 0.07 and 0.05 deer per ha at El Sauz, and 0.33 and 0.13 deer per ha at Santa Rosa. Nilgai estimates were similar between the sites, 0.11 and 0.17 nilgai per ha at El Sauz and 0.11 and 0.11 nilgai per ha at Santa Rosa for 2018 and 2019, respectively. Wild pig and collared peccary population estimates were combined in these surveys; El Sauz had 0.03 and 0.01 wild pig and collared peccary per ha, while Santa Rosa had 0.07 and 0.33 wild pig and collared peccary per ha, for 2018 and 2019, respectively.

## 3 MATERIALS AND METHODS

### 3.1 Fence description and condition

We surveyed boundary net‐wire fence lines at both sites to verify the presence of intact, maintained fences, with ≤7 cm between the bottom fence wire and the ground. We randomly selected a 9146‐m boundary fence at El Sauz and a 2174‐m boundary fence at Santa Rosa; fence lengths differed because of the configuration of the property boundaries. Boundary fences were selected over interior fences because they often form long, linear features with no openings (e.g., gates). Therefore, animals must go under or over the wire to pass beyond the fence. Both fence lines were standard net‐wire livestock fences 1.25 m in height. Both fences had an unpaved 2‐track road on both sides, with mesquite and huisache woodlands beyond the roads, except for the exterior side of the fence at Santa Rosa which was grassland. We drove a utility vehicle along target fence lines at each study site to identify and record fence‐crossing locations. At each identified crossing location, we recorded the maximum height of the bottom wire (m), and width (m) of each opening. We conducted these surveys of fence‐crossing locations during Autumn (October–November) 2017, 2018, and Spring (April–early June) 2018, 2019. We then calculated the opening size of each crossing (m^2^) as the maximum height multiplied by width. When fence crossings become large enough for livestock to pass through, a common practice at these study sites is to patch the hole by securing a panel of net‐wire livestock fence over the opening to discourage further crossings. Therefore, we also recorded fence‐crossing locations in relation to previous repairs or patched locations.

### 3.2 Landscape features

Landscape features can influence wildlife habitat use (Thogmartin, 2001; Van Dorp & Opdam, 1987; Zemanova et al., 2017), and thus may influence where animals choose to cross fences. We quantified woody cover at fence‐crossing locations using a spatial pattern analysis in ArcGIS ArcMap 10.5.1 (ESRI©, Redlands, CA) FRAGSTATS 4.2 (McGarigal et al., 2012) based on high‐resolution (1‐m) aerial multispectral images from the National Agriculture Imagery Program (NAIP) for 2016. We first classified imagery into 4 land cover types: woody cover, herbaceous, bare ground, and water using unsupervised image classification in ERDAS Imagine 2018 (Hexagon Geospatial; Xie et al., 2008). We conducted an accuracy assessment with 200 random points per image until ≥85% accuracy was achieved (Jensen, 2016; Pulighe et al., 2016). We created 30‐m buffers at fence‐crossing locations and at an equal number of random locations on the same fence line at both sites. We focused on the woody cover, as the most common cover types were woody and herbaceous; there was no permanent water near the boundary fencing, and the bare ground was sporadic and ephemeral. At El Sauz, random locations were adjusted to not overlap other known or random crossing buffers. This approach was not feasible in Santa Rosa because crossings were relatively abundant. We clipped the imagery to the extent of the buffers to quantify the amount and spatial structure of woody cover within buffer areas. We characterized woody cover using 6 landscape metrics (McGarigal et al., 2012): patch density (PD, number of woody patches/100 ha), percentage of the landscape in woody cover (PLAND %), the mean area of woody patches (AREA_MN), the Euclidean nearest‐neighbor distance between woody patches (ENN, m), the aggregation index (AI, frequency which like patches appear side by side, %) and edge density (ED, edge length of woody cover patches per unit area, m/ha).

### 3.3 Crossing‐site usage

To assess the usage of each crossing location at each study site, we randomly assigned 10 camera traps to fence crossings identified through the fence surveys (Reconyx© HyperFire HC500 or XR6 UltraFire, Reconyx, Holmen, WI; Moultrie© A‐5 Gen2 MCG‐12688 Moultrie feeders). We fastened cameras onto 1.5‐m metal t‐posts at a mean height (±SE) above ground of 0.54 ± 0.02 m (range 0.43–0.63) at El Sauz, and 0.66 ± 0.03 m (range 0.40–0.80) at Santa Rosa. The mean distance (±SE) from the t‐post to the crossing was 3.00 ± 0.12 m (range 2.40–3.58) at El Sauz and 1.72 ± 0.15 m (range 1.04–2.80) at Santa Rosa. The boundary fence at Santa Rosa often had an unpaved 2‐track road close to the fence and we could not place cameras on the road; thus, the distance between the crossing and the site of camera placement was shorter than for El Sauz. We placed the cameras higher up to angle down at the crossings to address the reduced distances between cameras and fence crossings. The cameras were focused on crossing locations where wildlife crawled underneath the fencing. Depressions on the top wire of these fences were rare, so we did not assess jumps over the fence by deer or nilgai.

We first deployed cameras in January 2018 as a pilot study to assess camera placement and photo quality. During the pilot study, on March 28, 2018, two fence crossings were patched with a panel of livestock fencing at El Sauz. In response, we kept cameras at the two patched locations and added cameras to two active, un‐patched fence crossings. These two patched crossings (ID: EF24 & EF25) provided an opportunity to assess wildlife response to the blocking well‐established fence crossings. Both patched crossings were monitored from April 2018 to March 2019. We checked cameras every two weeks to ensure functionality as extreme heat greatly reduced battery life, and frequent rubbing of the cameras by cattle increased camera failure. We programmed cameras to take a 3‐photograph burst with a 10‐s delay (Moultrie) or 15‐s delay between triggers (Reconyx), with high motion detector sensitivity. The minimum delay interval for the Moultrie cameras was 10‐s with 1‐s between photo bursts. A no‐delay setting would minimize missed crossing attempts, but our delay was sufficient due to the open visibility on the opposite side of the fence and limited occurrences of large groups (besides turkeys) passing through the fences. During the camera checks, we also measured the height (m) and width (m) of each fence‐crossing location to record any changes during the study.

### 3.4 Data analysis

We used a Kolmogorov–Smirnov test to compare the distributions of each landscape metric between known fence‐crossing locations and random locations along the fence line, implemented via the R programming language (R Core Team, 2013). Known crossings included any crossing location that was recorded during the four surveys. Multiple factors likely influence the distribution of fence crossings, and certain landscape features might promote clusters of fence crossings in areas. To understand whether fence crossings were randomly spaced or clustered across the fence lines we conducted a Wilcoxon signed rank test to compare distances between known crossings sites and distances between random sites along the fence.

We classified the first two weeks (336 h) of photographs each month per camera, from April 2018 to March 2019. We classified all animal events by species, time of day, date, and outcome of each attempted crossing event as successful or unsuccessful. A successful crossing event was an attempted crossing event where the 3‐photo burst showed an animal passing under the fence or had at least half of the body through the fence crossing. We classified “attempted crossing events” as animals in close proximity to the crossings, either between the camera and crossing (about 3 m), or on the opposite side of the fence that approached or came into contact with the fence. Attempted crossing events included animals that successfully crossed or had no resulting photos to verify a successful crossing event. Animals that clearly disregarded the fence (i.e., browsing or walking a trail nearby with photos of it walking in and out of the camera frame) were recorded but were not used in this analysis. If we had consecutive photo bursts of the same individual animal, it was not re‐counted, unless it was present for >1 min since classification. We classified unrecognizable photographs of animals as “unknowns,” and further categorized as unknown carnivore or unknown ungulates, when possible.

We hypothesized that wildlife crossing events could be influenced by vegetation composition in the vicinity of the site and characteristics of the fence (e.g., height and width of the opening). We first conducted diagnostics to evaluate the potential for multicollinearity and nonconstant variance. Preliminary analyses revealed that some of the 6 Fragstats metrics of the fence crossings were correlated (Table 1 and Table S1). Therefore, we conducted a principal components analysis on the standardized variables (mean of 0 and SD of 1) to reduce the dimensionality of the woody cover data and retain most of the variation in a reduced set of uncorrelated variables. We retained the top two principal components that explained most of the variation in the 6 woody cover metrics for further analyses. For fence characteristics, we used the size (height × width in m) of the opening in the fence and the proximity (distance in m) to the nearest crossing location to represent ease of crossing and the density of crossings (alternative sites to cross if unsuccessful) present.

**TABLE 1:** Means, medians, and range of 6 Fragstats variables generated from 30‐m buffers around 21 monitored fence crossings on two sites in South Texas, 2018–2019.

Variable^a^	Mean	Median	Range
PLAND	44.43	46.58	0–72.56
PD	7017	5686	0–19,483
ED	2230	2205	0–3531
AREA_MN	0.01	0.01	0–0.03
ENN_MN	2.74	2.68	0–5.03
AI	80.36	85.14	0–96.33

^a^ Percentage of the landscape in woody cover (PLAND %), patch density (PD, number of woody patches/100 ha), edge density, the edge length of woody cover patches per unit area (ED, m/ha),, the mean area of woody patches (AREA_MN), the Euclidean nearest‐neighbor distance between woody patches (ENN, m), the aggregation index, and the frequency which like patches appear side by side (AI, %).

We conducted generalized logistic regression analyses to model the probability of successful crossing events relative to biotic and abiotic covariates. We conducted separate analyses of crossing events by animal species under the assumption that species' body size and behavioral characteristics may influence the probability of successful crossing. We only analyzed species with sufficient detections to be informative: deer, nilgai antelope, collared peccary, wild pig, and coyote (*Canis latrans*). The final logistic regression model for each species included the binomial response (0 = unsuccessful crossing, 1 = successful crossing) and principal components of woody cover metrics, size of the opening, and distance to the nearest crossing as predictors; all predictors were standardized with a mean of 0 and a standard deviation of 1. We also included a season in the model because preliminary analyses revealed that the frequency of photographs was higher during winter, which indicated that the probability of crossing may vary seasonally. To quantify the potential season effect, we classified June, July, and August photographs as ‘summer’ and December, January, and February photograph as ‘winter’. These two seasons were included as a categorical variable in the final model to focus on relative hot and cool times of the year which may affect crossing rates as it relates to thermoregulation. Lastly, to account for potential spatial autocorrelation, we included fence crossing ID as a random effect in the models.

We conducted an additional suite of analyses aimed at understanding how characteristics of a crossing location may influence the number of crossing events and species that attempt to cross. For instance, are crossing‐site characteristics associated with use by more individuals or species, and so on. To understand the temporal activity pattern of crossing attempts by multiple species, we categorized time into 8 parts of the day, each 3 h in duration, starting with 05:00–07:59 h, since 05:00 h best encompassed dawn or the first hour of light during this study. We excluded species with <100 crossing attempts from this analysis, due to low occurrence. We calculated frequencies of crossing attempts per species, location, and time, and season. We quantified species diversity and richness, excluding cattle and unidentified animals, to account for both abundance and species evenness among crossing locations based on the Shannon‐Weiner index (Shannon, 1948). We also modeled the Shannon‐Weiner Index relative to the woody cover principal components, size of the opening, and nearest crossing via generalized linear models to determine if characteristics of crossing locations influenced the number and diversity of species that used the site. Principal component and regression analyses were conducted with R packages factoextra (Kassambara & Mundt, 2020), and lme4 (Bates et al., 2014).

## 4 RESULTS

### 4.1 Density, size, and placement of fence crossings

We detected 34 fence‐crossing locations (1 crossing/269 m) and 30 patched crossings (1 patch/305 m) from Autumn 2017 to Spring 2019 at El Sauz. The El Sauz average crossing location height (±SD) = 0.44 ± 0.13 m (range 0.18–1.00), width = 0.71 ± 0.26 m (range 0.21–1.70), and opening size = 0.32 ± 0.29 m^2^ (range 0.06–1.09). Over half of the crossing locations (53%) were adjacent to a previously patched crossing. At Santa Rosa, we detected 52 fence‐crossing locations (1 crossing/42 m) and 2 patched crossings (1 patch/1087 m). One crossing was patched in December 2018, and the other patch was first recorded in the Autumn 2018 survey next to a crossing. At Santa Rosa, the average crossing location height (±SD) = 0.61 ± 0.13 m (range 0.31–0.94), width = 1.10 ± 0.44 m (range 0.33–3.00), and opening size = 0.69 ± 0.36 m^2^ (range 0.15–2.61). Our randomly selected fence crossings monitored via cameras had similar dimensions. For the 10 crossings monitored with cameras at El Sauz, the mean height (±SD) = 0.44 ± 0.09 m (range 0.32–0.60), width = 0.68 ± 0.11 m (range 0.52–0.93), and size = 0.30 ± 0.09 m^2^ (range 0.18–0.43). The 10 crossings monitored at Santa Rosa had a mean height = 0.59 ± 0.11 m (range 0.35–0.73), width = 1.22 ± 0.33 m (0.79–1.67), size = 0.75 ± 2.91 m^2^ (0.26–1.37).

We found no statistical differences between crossings and random locations for any of the land cover metrics at 30‐m buffers (Table 2; Kolmogorov–Smirnov Z *p* values > .58 on Santa Rosa and >0.84 on El Sauz). The mean distance between crossing points and distance between random points on El Sauz were similar (271.3 m crossings, 270.6 m random). The mean distance between crossing points and distance between random points at Santa Rosa was slightly higher at crossings (43.5 m crossings, 40.9 m random). There was no difference between median distance of crossing locations and random locations (El Sauz Wilcoxon = 519, critical value = 159, *p* = .93; Santa Rosa Wilcoxon = 1183, critical value = 453, *p* = .44).

**TABLE 2:** Comparison of values for 6 woody vegetation metrics obtained from 30‐m buffers around crossings and random locations on El Sauz and Santa Rosa in South Texas, 2018–2019. Sample sizes varied on El Sauz because some of the patch‐related Fragstats variables for certain fence locations were unable to be generated.

Variable^a^	Location	El Sauz			Santa Rosa		
		N	Value	SE	N	Value	SE
AI	Crossing	33	70.5	5.96	52	84.1	0.57
AI	Random	33	75.0	5.01	52	83.7	0.68
AREA_MN	Crossing	33	0.01	0.00	52	8.1	0.75
AREA_MN	Random	33	0.01	0.00	52	8.7	0.94
ED	Crossing	33	1285.8	170.07	52	26.6	0.69
ED	Random	33	1455.0	170.22	52	27.2	0.71
ENN_MN	Crossing	33	3.7	0.86	52	2.8	0.05
ENN_MN	Random	33	3.2	0.39	52	2.8	0.06
PD	Crossing	33	4508.7	690.09	52	69.3	3.89
PD	Random	33	4356.6	650.42	52	70.9	4.14
PLAND	Crossing	33	36.8	4.94	52	44.2	1.65
PLAND	Random	33	37.1	4.81	52	45.2	1.81

^a^ Percentage of the landscape in woody cover (PLAND %), edge density, the edge length of woody cover patches per unit area (ED, m/ha), patch density (PD, number of woody patches/100 ha), the mean area of woody patches (AREA_MN), the Euclidean nearest‐neighbor distance between woody patches (ENN, m), the aggregation index, and the frequency which like patches appear side by side (AI, %).

### 4.2 Animal behavior and usage

Both sites had photographs of armadillo (*Dasypus novemcinctus*), bobcat (*Lynx rufus*), domestic cattle, coyote, deer, wild pig, collared peccary, nilgai, raccoon (*Procyon lotor*), striped skunk (*Mephitis mephitis*), and turkey. We detected an additional 4 species at El Sauz, including badger (*Taxidea taxus*), ocelot (*Leopardus pardalis*), and lagomorphs (*Sylvilagus floridanus*, *Lepus californicus*); we observed but did not classify rodents. With the exception of turkey, birds were classified but not included in this analysis because most were photographs of perching birds on the fence and other small birds that are not dependent on crossing locations, such as roadrunners (*Geococcyx californianus*) and northern bobwhite (*Colinus virginianus*). Deer had the most crossing attempts at both sites (44% El Sauz, 58% Santa Rosa), followed by nilgai (14% El Sauz, 8% Santa Rosa). Deer and wild pigs successfully crossed at all 20 fence crossings monitored. Most fence crossings had ≥1 successful crossing by collared peccary (95% of monitored fence‐crossing locations at both sites), coyotes (90%), bobcats (67%), nilgai (71%), and raccoons (52%).

**FIGURE 5:**
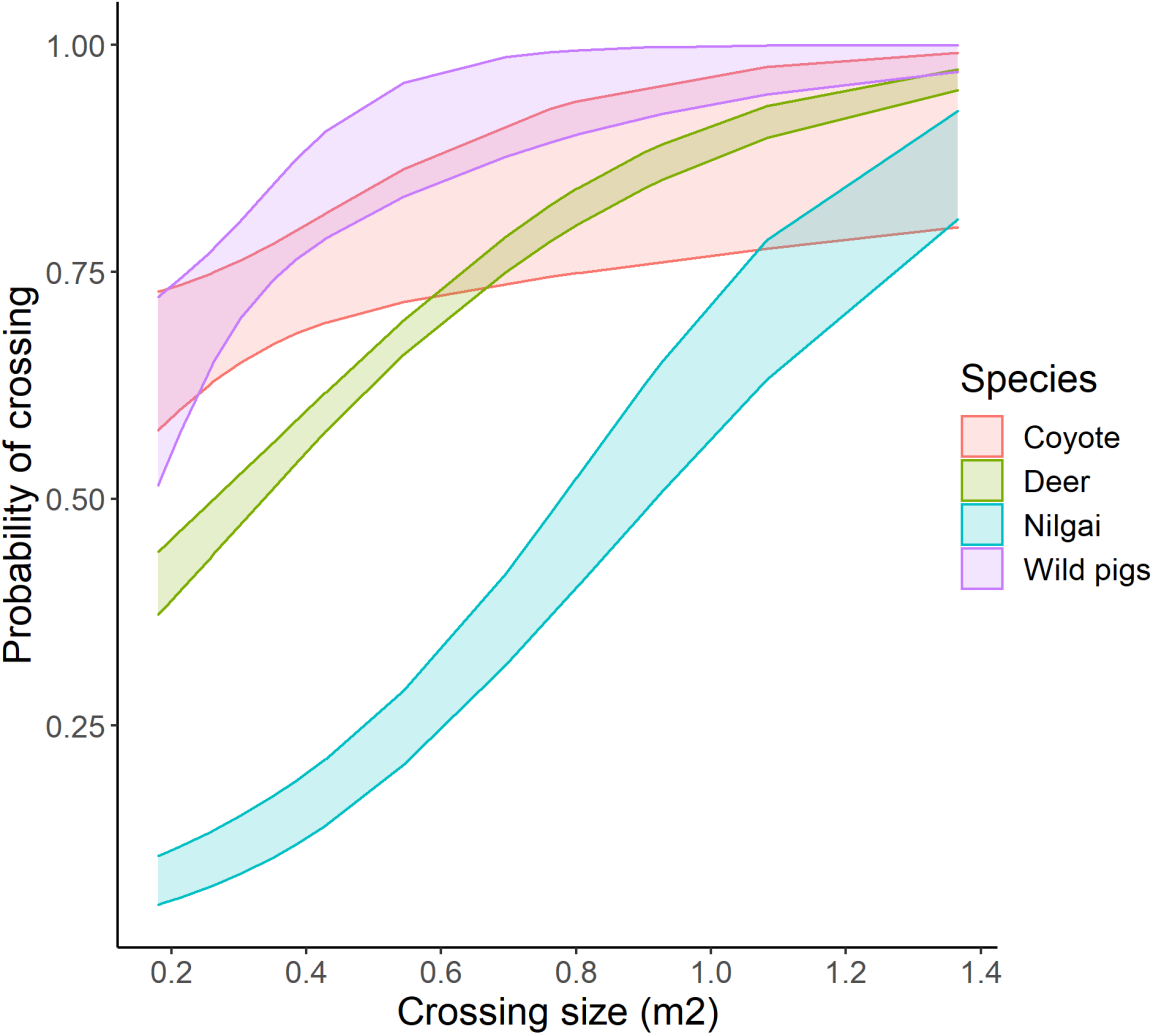
Species‐specific probability of crossing in relation to crossing size (m^2^) at fence lines with 95% CI in South Texas, USA during 2018–2019.

Our principal components analyses revealed two dimensions that totaled 85.5% of the variation in woody vegetation (Table 3); we used those two principal components in our logistic regression models. Our logistic regression models for the 5 species revealed that the probability of crossing was most consistently influenced by crossing size and season (Table 4). As crossing size increased, the probability of crossing significantly increased for all species except collared peccary. Further, the probability of crossing differed among species (Figure 5). The lowest height recorded for successful crossing by deer was 32 cm and the smallest size was 0.18 m^2^. Nilgai successfully crossed at 7 of 10 crossings at El Sauz. The 3 crossings with no successful crosses from nilgai were 33–34 cm in height and 0.15–0.23 m^2^ in size. At Santa Rosa, nilgai successfully crossed at 8 of the 11 crossings monitored. The crossings nilgai did not successfully cross ranged from 35–52 cm in height and 0.26–0.80 m^2^ in size. The lowest height for nilgai success overall was 44 cm and the smallest size was 0.26 m^2^. All species except for collared peccary had a significantly higher probability of crossing during summer months relative to winter months (Table 4). Coyotes appeared to have the highest differential relative to other species; the probability of crossing was very low during winter months. Across species, the probability of crossing did not appear to be consistently influenced by woody characteristics or distance to the nearest crossing.

**TABLE 3:** Statistics and variables associated with principal component (PC) analyses of woody vegetation characteristics at fence crossing locations on rangelands in South Texas, USA, during 2018–2019. Principal components 3 to 6 are not shown (total 14.5% of variance explained).

Variable^a^	PC 1^b^	PC 2^c^
PLAND	0.44	0.48
PD	0.76	0.00
ED	0.56	0.27
AREA	0.80	0.02
ENN	0.10	0.77
AI	0.92	0.00

^a^ Percentage of the landscape in woody cover (PLAND %), edge density, the edge length of woody cover patches per unit area (ED, m/ha), patch density (PD, number of woody patches/100 ha), the mean area of woody patches (AREA_MN), the Euclidean nearest‐neighbor distance between woody patches (ENN, m), the aggregation index, and the frequency which like patches appear side by side (AI, %).

^b^ Eigenvalue = 3.57 and 59.7% of variance explained.

^c^ Eigenvalue = 1.55 and 25.8% of variance explained.

**TABLE 4:** Factors influencing probability of crossing (SE) for the 5 species most frequently photographed at fence crossings in South Texas, 2018–2019.

Species	PC 1^a^	PC 2^b^	Size^c^	Nearest^d^	Season‐Winter^e^
White‐tailed deer	0.33 (0.09)*	−0.11 (0.13)	0.70 (0.19)*	−0.32 (0.19)	−0.58 (0.09)*
Nilgai	0.18 (0.17)	−0.00 (0.25)	1.71 (0.44)	0.41 (0.37)	−0.63 (0.25)*
Wild pig	0.06 (0.12)	0.26 (0.13)	1.83 (0.67)*	−0.13 (0.23)	−1.11 (0.32)*
Coyote	0.07 (0.07)	−0.33 (0.15)*	0.65 (0.29)*	−0.16 (0.22)	−2.06 (0.38)*
Collared peccary	0.20 (0.14)	−0.18 (0.21)	0.19 (0.48)	−0.96 (0.48)*	−0.67 (0.48)

^a^ Principal component (PC) analysis of woody vegetation characteristics with Eigenvalue = 3.57 and 59.7% of variance explained.

^b^ Principal component analysis of woody vegetation characteristics with Eigenvalue = 1.55 and 25.8% of variance explained.

^c^ Size of opening of the crossings.

^d^ Distance to nearest crossing.

^e^ In reference to summer.

* Denotes statistically significant at *p* ≤ .05.

The fence crossings at El Sauz had 6229 attempted crossings with 50% success (*n* = 3128), from 37,822 h monitored (Table 5). At Santa Rosa, there were 4660 attempts with 67% success (*n* = 3143) from 35,763 h monitored (Table 5). El Sauz and Santa Rosa, respectively, averaged 4.0 and 3.1 crossing attempts/day, and 2.0 and 2.1 successful events/day (Table 3). El Sauz had attempts ranging from 127 (1.1/day) −1289 (7.8/day) per monitored crossing. Santa Rosa crossing locations had attempts ranging from 260 (1.7/day) −759 (5.2/day). Successful events/day for nilgai were similar between El Sauz (0.09, *n* = 140) and Santa Rosa (0.13, *n* = 190). Deer successfully crossed at Santa Rosa (1.43, *n* = 2143) more often than at El Sauz (0.87, *n* = 1376), similar to differences in population density. Wild pigs had higher frequencies of successful crossing at El Sauz (0.36, *n* = 566) than Santa Rosa (0.17, *n* = 248), and frequencies of successful crossing by collared peccary were similar at El Sauz (0.12, *n* = 183) and Santa Rosa (0.17, *n* = 249).

**TABLE 5:** Fence‐crossing events and frequencies by species during April 2018–march 2019 as recorded by remote cameras at 10 fence crossing‐locations on the El Sauz and Santa Rosa ranches, Willacy and Kenedy counties, South Texas, USA.

Species	Attempted crossings (successful^a^)		Mean attempts/day (successful^a^)	
	El Sauz	Santa Rosa	El Sauz	Santa Rosa
Armadillo	23 (15)	1 (1)	0.015 (0.010)	0.001 (0.001)
Badger	3 (3)	0 (0)	0.002 (0.002)	0.000 (0.000)
Bobcat	100 (88)	9 (9)	0.063 (0.056)	0.006 (0.006)
Cattle	372 (0)	615 (7)	0.236 (0.000)	0.413 (0.005)
Collared peccary	272 (183)	280 (249)	0.173 (0.116)	0.188 (0.167)
Coyote	419 (301)	144 (120)	0.266 (0.191)	0.097 (0.081)
White‐tailed deer	2738 (1376)	2690 (2143)	1.737 (0.873)	1.805 (1.438)
Lagomorph	51 (13)	0 (0)	0.032 (0.008)	0.000 (0.000)
Nilgai	861 (140)	379 (190)	0.546 (0.089)	0.254 (0.128)
Ocelot	4 (3)	0 (0)	0.003 (0.002)	0.000 (0.000)
Raccoon	66 (42)	10 (6)	0.042 (0.027)	0.007 (0.008)
Skunk	3 (0)	14 (12)	0.002 (0.001)	0.009 (0.008)
Small rodent	1 (1)	0 (0)	0.001 (0.246)	0.000 (0.000)
Turkey	522 (388)	233 (155)	0.331 (0.005)	0.156 (0.104)
Wild pig	776 (566)	274 (248)	0.492 (0.359)	0.184 (0.166)
Unknown	14 (8)	7 (3)	0.009 (0.005)	0.005 (0.002)
Unknown carnivore	2 (1)	0 (0)	0.001 (0.001)	0.000 (0.000)
Unknown ungulate	2 (0)	4 (0)	0.001 (0.000)	0.003 (0.000)
Total	6229 (3128)	4660 (3143)	3.953 (1.985)	3.127 (2.109)

^a^ Photo of an animal passing through fence crossing or had at least half of the body through.

Total species richness and diversity for El Sauz and Santa Rosa were 14 vs. 10 species, and the overall Shannon–Weiner index of diversity was 1.65 versus 1.19, respectively. Our logistic regression with variables of principal components of woody cover metrics, size of opening, distance to nearest crossing, and season, indicated that crossing size was the only variable significantly influencing Shannon–Weiner index of diversity (*β* = −0.25 ± 0.11 SE, *p* = .03); as crossing size increased, diversity of species that attempted to cross declined. Because the negative beta was counterintuitive, we conducted post‐hoc analyses on species richness or the number of unique species photographed at each crossing. Species richness was not statistically influenced by any of the 4 covariates we tested (*p* > .33); thus, the negative correlation between diversity (which is weighted by the number of photographs at each crossing, Supp. Info Table 1) and crossing size was attributed to the increase in the frequency of crossings of deer and nilgai as the size of the opening increased.

### 4.3 Response to repair of fence crossings

**FIGURE 6:**
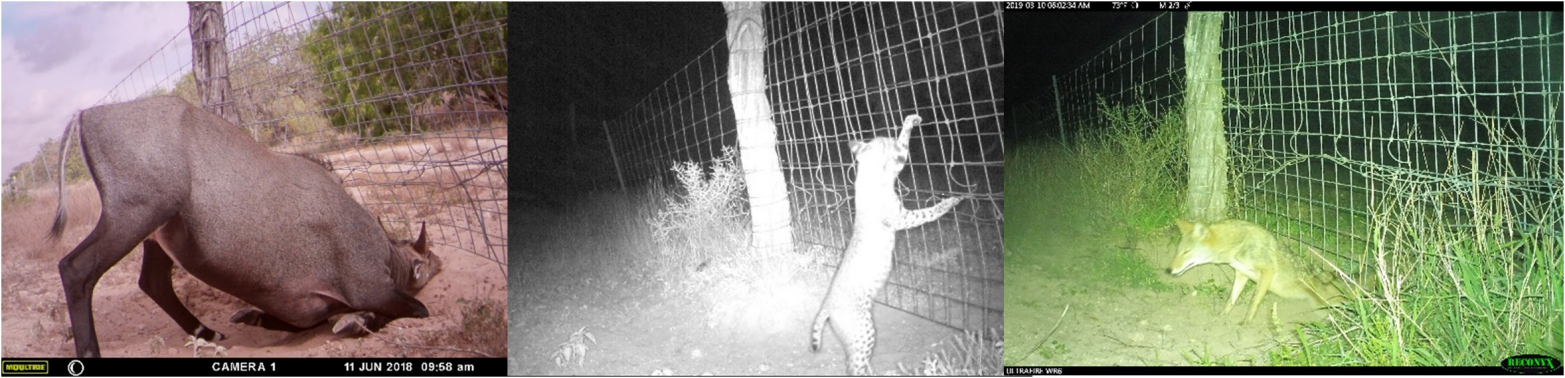
A nilgai antelope bull attempts to push through a patched crossing in a typical crossing stance, a bobcat attempts to climb the patch, and a coyote crawls beneath the patch at the El Sauz ranch in Willacy County, South Texas, USA, 2018–2019. When crossings become enlarged to the point of damage to the fence or allow livestock to escape, ranches repair, and reinforce the fence. However, nilgai and other animals often persistently attempt to cross at the same location or nearby.

We recorded no successful crossing events from deer and nilgai at the two patched locations while the patches were intact; crossing heights of both locations were 20 cm, and sizes = 0.14 m^2^ and 0.20 m^2^. One of the patched crossings (EF24) remained patched until August 3, 2018, when a nilgai bull pushed open the panel, which returned the fence‐crossing to its' original size. This provided documentation of fence‐crossing re‐establishment. While the location was patched, bobcat, turkey, coyotes, and collared peccaries successfully crossed. Average frequency of attempts of all animals (attempts/day) for EF24 was 22% lower when patched (5.1, *n* = 285) than when opened (6.5, *n* = 640). The frequency of successful crosses (events/day) was lower when patched (0.2, *n* = 11) than when opened (4.6, *n* = 446). Patched crossing EF25 remained undamaged throughout the study period. Patched EF25 had successful events from 1 coyote, 4 wild pigs, and 2 collared peccaries, and averaged 1.41 attempts/day, *n* = 237. We recorded deer and nilgai pushing their heads under the patch, a bobcat and coyotes digging beneath the patch, and a bobcat climbing over the fence at the patch location (Figure 6). A fence crossing at Santa Rosa was patched in December 2018; this camera was removed and placed on a new fence crossing to continue the evaluation of fence crossing use. Additional cameras were not available to investigate this patched location. Before the crossing at Santa Rosa was patched, it had high use (4.4 attempts/day, 490 attempts) for the 8 months monitored, and was the largest crossing (1.37 m^2^).

## 5 DISCUSSION

Infrastructure is constantly increasing on the landscape, leading to impacts on wildlife worldwide (Forman, 2000; Jaeger & Fahrig, 2004; Jakes et al., 2018; Torres et al., 2016). This expansion has brought an increased need to understand the effects of anthropogenic features on wildlife. Fences are a common part of the landscape in southwestern rangelands. Most previous research on fence crossings only focused on a single or few ungulate species and excluded other animals from the analysis (Jakes et al., 2018; McInturff et al., 2020). In many cases, net‐wire livestock fencing is constructed for the sole purpose of enclosing cattle, but many large southwestern rangelands manage for wildlife as well. Our study confirms that fences clearly affect animal movement and behavior.

There was no apparent pattern in the location of fence crossings on the landscape; fence crossing locations did not differ from random locations. We also found no differences between woody cover, patch density, edge density, and distance of fence‐crossing locations compared to random locations within 30‐m buffers. It is possible that the location of crossings may be associated with finer or broader‐scale features than assessed by the 30‐m buffers. Alternatively, if multiple species created the crossings, each species may have different preferences for the location. Finally, the fence crossing location may simply be a good location to cross, which may be more associated with characteristics of the fence, fence condition, and soil substrate, rather than habitat features.

We detected 15 species of medium‐ and large‐size mammals and turkey to use crossings, representing all common medium‐ and large‐size mammal species known to be present on the two ranches. Crossings were used during all times of the day, and activity patterns were similar within‐species between sites. At El Sauz, there were peaks in crossing activity during May and between December–January for coyotes, deer, wild pigs, collared peccary, nilgai, and turkey (Zoromski, 2019). These species at Santa Rosa, besides wild pig, peaked from April–July and between December–February. The activity of wild pigs peaked in June and September at Santa Rosa (Zoromski, 2019). Overall daily activity patterns for attempts were similar during the morning, afternoon, and night, each contributing 21%–30% of the total visitations (Zoromski, 2019). Dawn consisted of the lowest percentage (9% for both sites), followed by dusk (13% El Sauz, 17% Santa Rosa; Zoromski, 2019). These activity patterns are consistent with normal peaks of activity for each species (Zoromski, 2019). We observed crossing peaks during May and December–February for many species on both sites. The peaks in winter may be attributed to the rutting behavior of large mammals (nilgai: Fall, 1972, Sheffield et al., 1983, deer: Foley et al., 2015). In both cases, crossing activity corresponded with a typical increase in daily and seasonal movements.

Although many species used fence crossings, there was evidence that net‐wire livestock fencing may act as a partial or complete barrier to movement, dependent on the species considered. First, 30%–50% of attempted crossings were unsuccessful, evidence that fences affected animal movements, and behavior. As crossing size increased, the probability of crossing significantly increased for all species. This finding is similar to previous studies, which found the number of crossing attempts increased for some species after modifications to existing fences, such as the addition of smooth (non‐barbed) bottom wire or clips to elevate the bottom wire (Burkholder et al., 2018; Jones et al., 2018, 2020). These wildlife‐friendly modifications with elevated bottom wire and smooth wire may also reduce the time for species to cross and increase the probability of successful crossings of wildlife, and effectively enclose livestock (Segar & Keane, 2020). We occasionally observed deer and nilgai use fence crossings at a full sprint; it seems likely the animals were familiar with and had previously used the crossing locations. We were not able to identify individuals, but some animals were recognizable using the same crossings throughout the year (e.g., wild pig with distinctive spot patterns, or antlered deer). Finally, many animals that were small enough to fit through the fence mesh still chose to use fence crossings. For instance, bobcats and raccoons can fit through 31 × 20 cm mesh fencing, but use crossings often.

We observed minor differences in species and frequencies of wildlife using fence crossings between sites. Overall, El Sauz had fewer crossing locations, higher rates of crossing attempts on average, greater wildlife diversity, and a higher Shannon's diversity index than Santa Rosa. Both sites had a similar percentage of successful crossings. Fencing at El Sauz was newer and better‐maintained, which resulted in fewer crossing locations. This may have funneled more animals through the available locations than at Santa Rosa, where crossings were more abundant. Overall, rates of successful fence crossing attempts corresponded to population densities estimated through aerial surveys for nilgai and deer. While many factors influence crossing rates, this indicates that fence crossing rates could be related to animal densities.

When crossings became vulnerable to cattle passage, landowners patched the fence crossings. To our knowledge, wildlife behavioral response to patched pre‐established crossings had never been studied in southwestern rangelands. While patching fence crossings is important to maintain fence integrity, wildlife often creates new crossings adjacent to these patched crossings. Although we only studied two patched crossings, these patches impeded deer and nilgai movements resulting in no successful crossing events. The patching did not limit all species. Bobcats, turkey, coyote, collared peccary, and wild pigs successfully crossed the patched crossings. These species could have crossed along other stretches of the fence but chose to cross at the patch site. Animals still attempted to cross at these locations, suggesting strong site preference. Over half (53%) of the fence crossings at El Sauz were adjacent to patched locations. We recorded photographs of nilgai and deer pushing their heads under the patch, bobcats and coyotes digging beneath; a bobcat climbed over the patched fence after unsuccessful crossing attempts. These attempts to cross‐patched fencing often led to additional damage to the fence. Barriers to wildlife movements have been shown to increase energy expenditure for animals that try to avoid or traverse the barrier (Buchanan et al., 2014; Dyer et al., 2001; Jacobson et al., 2016; Sawyer et al., 2009) and increase escape time (Hölzenbein & Marchinton, 1992; ZhangQiang et al., 2013). If an animal preferences a particular crossing location and if that crossing location were to be removed (patched), it may impede its' escape time or increase energy expenditure to find other crossing locations.

To our knowledge, this is the first study to recognize the importance of fence crossing locations for wildlife movement at a community level in southwestern rangelands. Despite our limited study period, we recorded over 10,000 crossing attempts, with 3–4 crossing attempts per day on average. This extrapolates to hundreds of crossings attempts beneath fences per day on these large sites, especially considering the sites have more fenced areas than the 11.3 km of fencing we monitored. In addition to effects on animal movement, crossing locations also concentrate animals and enable movement between adjacent properties. The fence crossings monitored at both sites revealed visitation from both domestic and wild animals, including invasive species. Therefore, fence crossings may be important locations for monitoring and controlling disease spread. Monitoring contact rates through fences is a key component of control measures for diseases, including chronic wasting disease and bovine tuberculosis (Fischer et al., 2011; Lavelle et al., 2010; Mysterud & Rolandsen, 2018; Vercauteren et al., 2007). Repairs to crossings may be a temporary solution since many animals often ruin the patch or damage the fence near it. Landowners could consider fence modifications, such as metal posts that limit the width size of a crossing or a horizontal metal bar at the desired fence crossing opening size to reduce damage yet allow wildlife to cross. Alternatively, leaving fence crossings can benefit wildlife while still enclosing cattle until the opening becomes too large. While animals may have alternative ways of crossing fences, the fence‐crossing locations were used by all common and rare mammal species and turkeys for movement.

## AUTHOR CONTRIBUTIONS

**Lisa Diane Zoromski:** Formal analysis (lead); investigation (lead); methodology (equal); project administration (lead). **Randy DeYoung:** Supervision (lead); writing – review and editing (equal). **John A. Goolsby:** Funding acquisition (lead); resources (equal). **Aaron M. Foley:** Conceptualization (supporting); formal analysis (equal); writing – review and editing (equal). **J. Alfonso Ortega‐Santos:** Conceptualization (supporting); resources (equal). **David G. Hewitt:** Conceptualization (supporting); resources (equal). **Tyler Campbell:** Conceptualization (supporting); resources (equal).

## FUNDING INFORMATION

Funding would be from the Las Huellas Organization of South Texas.

## CONFLICT OF INTEREST

The authors have no conflicts of interest.

## Supporting information


Table S1

## Data Availability

The data has been archived at Dryad with https://doi.org/10.5061/dryad.n8pk0p2zs.
