# Supplementary material for: Animal use of fence crossings in southwestern rangelands
Source: Ecol Evol. 2022 Oct 1;12(10):e9376. doi: 10.1002/ece3.9376 (PMC9526119; doi:10.1002/ece3.9376)
Supplement: Supplementary file 1 — Table S1 [file ECE3-12-e9376-s001.pdf]

	PLAND	PD	ED	AREA_MN	ENN_MN	AI
PLAND		-0.02	0.5	0.67	-0.02	0.83
PD	-0.02		0.6	-0.5	-0.08	0.23
ED	0.5	0.6		-0.09	-0.08	0.54
AREA_MN	0.67	-0.5	-0.09		-0.14	0.5
ENN_MN	-0.02	0.08	-0.08	0.14		0.36
AI	0.83	0.23	0.54	0.5	0.36	
